# *Malus pumila* Mill. cv Annurca apple extract might be therapeutically useful against oxidative stress and patterned hair loss

**DOI:** 10.1002/2211-5463.13805

**Published:** 2024-05-06

**Authors:** Nadia Benedetto, Claudia Mangieri, Filomena De Biasio, Ricardo Filipe Carvalho, Luigi Milella, Daniela Russo

**Affiliations:** ^1^ Department of Science University of Basilicata Potenza Italy; ^2^ EVRA S.r.l. Società Benefit Lauria Italy; ^3^ Spinoff Bioactiplant S.r.l. Potenza Italy

**Keywords:** androgenetic alopecia, Annurca apple extract, antioxidant effect, nutraceutical products, patterned hair loss

## Abstract

Patterned hair loss (PHL) or androgenetic alopecia is a condition affecting about 50% of people worldwide. Several pharmacological medications have been developed over the years, but few studies have investigated their effectiveness. Therefore, new, safer and more effective strategies are required. Recent investigations showed that Annurca apple extract application could induce keratin production and promote hair growth thanks to the high amount of procyanidin B2 contained in. Hence, this study aimed to investigate the role of an Annurca apple extract in preventing PHL by testing it on human follicle dermal papilla cells (HFDPCs) for the first time. Treatment of HFDPCs with Annurca apple extract counteracted intracellular reactive oxygen species accumulation by increasing the activity of antioxidant enzymes such as superoxide dismutase 2 and catalase. Furthermore, treatment with Annurca apple extract increased β‐catenin and fibroblast growth factor 2, which are involved in hair growth stimulation. These data suggest that Annurca apple extract may be a potential therapeutically useful nutraceutical product for preventing or treating hair loss by reducing oxidative stress and inducing the expression of hair growth‐related factors.

## Graphical abstract

*Malus pumila* Mill. cv Annurca extract was tested on hair follicle dermal papilla cells, demonstrating its ability to reduce intracellular reactive oxygen species and to induce the expression of hair growth‐related factors. AT HAIR‐FUL AA^®^ may be a potential therapeutically useful natural substance for preventing or treating hair loss.

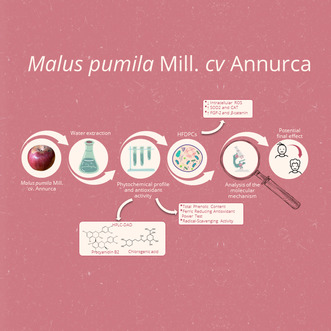

**Abbreviations**
5AR: 5‐α reductase enzyme
ABTS: 2,2′‐azino‐bis(3‐ethylbenzothiazoline‐6‐sulfonic acid) diammonium salt
CAT: catalase
CGA: chlorogenic acid
DCFH‐DA: 2′,7′‐dichlorodihydrofluorescein diacetate
DPPH: 2,2‐diphenyl‐2‐picryl hydrazyl radical
FDA: Food and Drug Administration
FeCl_3_ 6H_2_O: iron (III) chloride hexahydrate
FGF: fibroblast growth factors
FGF‐2: fibroblast growth factor 2
FIN: finasteride
FRAP: ferric reducing antioxidant power
H_2_O_2_
: hydrogen peroxide
HFDPCs: human follicle dermal papilla cells
HPLC‐DAD: high‐performance liquid chromatography‐diode array detector
mg GAE·g^−1^ DW: milligrams of gallic acid equivalent per gram of dried weight
MIN: minoxidil
MnSOD: manganese superoxide dismutase
MTT: 3‐(4,5‐dimethyl‐2‐thiazolyl)‐2,5‐diphenyl‐2H‐tetrazolium bromide dye
Na_2_CO_3_
: sodium carbonate
NAC: *N*‐acetyl‐l‐cysteine
PB2: procyanidin B2
PBS: phosphate‐buffered saline
PBS‐T: 0.025% Tween‐20 in PBS
PGI: protected geographic indication
PHL: patterned hair loss
ROS: reactive oxygen species
SD: standard deviation
SOD2: superoxide dismutase 2
TPC: total phenolic content
TPTZ: 2,4,6‐tripyridyl‐s‐triazine
Trolox: 6‐hydroxy‐2,5,7,8‐tetramethylchroman‐2‐carboxylic acid
UAE: ultrasound‐assisted extraction
VEGFA: vascular endothelial growth factor A
Wnt: Wingless/Integrated

Hair development is a cyclic process characterised by four phases: anagen (growth), catagen (regression), telogen (rest) and exogen (shedding). Anagen is the growth phase of the hair follicle cycle and can last from 2 to 6 years. Following this, the catagen phase is characterised by regression and involution of the hair bulb over approximately 2 weeks. About 85–90% of scalp hairs are in the anagen stage. The telogen phase is defined as the duration between complete follicular regression and the beginning of the subsequent anagen phase; it lasts 2–4 months and 10–15% of hairs are in this phase [1]. Knowing the hair follicle cycle is essential to understanding hair loss, as all causes of hair loss affect it in some manner [2]. The duration of each phase is influenced by a person's age, hormonal and nutritional status, and the position of the hair [1]. Adults typically lose between 200 and 300 hairs or 40 and 100 from their scalp each day, depending on whether they use shampoo or not [2]. A new hair grows in to replace each one that is lost; however, in some cases, there may be an interruption of growth, causing abnormal hair loss during the anagen phase (anagen effluvium). This can be a prelude to patterned hair loss (PHL) or androgenetic alopecia, a condition affecting about 50% of people worldwide, regardless of age and gender [3, 4]. Only two drugs have been authorised by the Food and Drug Administration (FDA) for the treatment of PHL: finasteride (FIN) and minoxidil (MIN). FIN is an inhibitor of the type II isoform of the 5‐α reductase enzyme (5AR), which causes an increase in dihydrotestosterone, thereby reducing the anagen phase and the progressive hair matrix cells' miniaturisation. In contrast, MIN appears to increase the hair dermal papillae follicular vascularity, lengthen the anagen phase and shorten the telogen phase by acting as a potassium channel opener [3, 5]. However, the activity of FIN and MIN reach a plateau within 2 years of use and both induce side effects in patients [6, 7]. For this reason, there is a demand for new therapeutic strategies. Particular attention has been paid recently to the role of nutritional and antioxidant therapy for managing hair loss since it has been demonstrated that dietary supplements enriched with vitamins, plant extracts and amino acids could increase the rate of anagen in PHL patients [3]. In this context, oligomeric procyanidins, isolated from grape seeds, have drawn attention in the pursuit of biological products with hair growth‐promoting activity because they can stimulate the growth of hair epithelial cells as well as induce anagen phase to almost the same extent as MIN [8]. Specifically, procyanidin B2 was shown to be among the safest and most effective compounds usable in inducing hair growth *in vitro* [9] and in humans when applied topically [10, 11]. Among all sources of procyanidin B2, apple fruits contained the highest concentration of this active metabolite. In particular, it has been reported that the cv Annurca produces the most oligomeric procyanidin and has the greatest content of procyanidin B2 in comparison to other commercial apples such as Granny Smith, Fuji, Red Delicious, Pink Lady and Golden Delicious [12]. With the present investigation, it was therefore decided to investigate the antioxidant activity and hair growth effect of ‘Annurca’ apple fruit (*Malus pumila* Miller cv Annurca). Annurca is an apple variety widely cultivated in the Campania region of southern Italy, known for its white flesh, crispness and acidic taste due to its high acid/sugar ratio. Annurca apple accounts for 5% of national and 60% of regional apple fruit production and a yearly income of 100 million euros [13]. This apple variety is characterised by unusual postharvest storage requirements. The fruit is not fully matured when it is harvested and is subjected to a particular treatment in traditional structures known as ‘melai’, which are characterised by a straw layer that supports the fruits and is coated with screening nets to limit solar radiation and temperature exposure. When the side facing the sun turns red, the apple is rotated to give its opposite side a chance to turn red [14]. The consumption of Annurca apple fresh fruit was demonstrated to reduce age‐associated alterations in rats by lowering the activity of reactive oxygen species (ROS), thus exerting an antioxidant effect [15]. Similarly, it has been reported that apple juice consumption increases human blood antioxidant levels [16]. Furthermore, a recent clinical trial demonstrated that Annurca apple decreased hair loss and increased hair weight and density [17]. For this reason, the present study aimed to investigate for the first time the potential antioxidant activity and hair growth effect of Annurca apple extract, ‘AT HAIR‐FUL AA^®^’, in order to validate its application in nutraceutical and cosmetic applications for the prevention of PHL.

## Materials and methods

### Chemicals

Folin–Ciocalteu reagent, 2,2‐diphenyl‐2‐picryl hydrazyl radical (DPPH), sodium carbonate (Na_2_CO_3_), 2,2′‐azino‐bis(3‐ethylbenzothiazoline‐6‐sulfonic acid) diammonium salt (ABTS), potassium persulfate, 2,4,6‐tripyridyl‐s‐triazine (TPTZ), iron (III) chloride hexahydrate (FeCl_3_ 6H_2_O), 6‐hydroxy‐2,5,7,8‐tetramethylchroman‐2‐carboxylic acid (Trolox), procyanidin B2 (PB2), chlorogenic acid (CGA), 2′,7′‐dichlorodihydrofluorescein diacetate (DCFH‐DA), *N*‐acetyl‐l‐cysteine (NAC), 3‐(4,5‐dimethyl‐2‐thiazolyl)‐2,5‐diphenyl‐2H‐tetrazolium bromide dye (MTT), hydrogen peroxide (H_2_O_2_), methanol, hydrochloric acid (HCl), dimethyl sulfoxide (DMSO), Bradford reagent and RIPA Buffer were purchased from Sigma (St. Louis, MO, USA and Steinheim, Germany).

### Extract preparation and procyanidin B2 and chlorogenic acid content

The Annurca apple whole fruit (peel and pulp) extract (AT HAIR‐FUL AA^®^) was obtained as described previously by De Biasio *et al*. [17]. The extract was manufactured by Evra S.r.l. (Lauria, Potenza, Italy).

The quantification of procyanidin B2 and chlorogenic acid was performed by high‐performance liquid chromatography‐diode array detector (HPLC‐DAD), as reported previously [17].

### Total phenolic content

The total phenolic content (TPC) was determined using the Folin–Ciocâlteu reagent. It is based on electron transfection, permitting the measurement of the antioxidants' reductive capacity. The extract (75 μL) was incubated with Folin–Ciocâlteu reagent (500 μL) and Na_2_CO_3_ (500 μL). Distilled water was added to reach a final volume of 1.5 mL. The reaction mixture was incubated for 1 h in the dark, and absorbance subsequently measured at 723 nm on a UV–VIS spectrophotometer (SPECTROstar Nano BMG Labtech, Ortenberg, Germany). The results are expressed as milligrams of gallic acid equivalent per gram of dried weight (mg GAE·g^−1^ DW) [18].

### 2,2‐Diphenyl‐1‐picrylhydrazyl

The DPPH assay was used to measure radical‐scavenging activity. It evaluates the reducing capacity of an extract against 2,2‐diphenyl‐1‐picrylhydrazyl, a stable free radical that has a deep purple colour in its oxidised form [19]. In a 96‐well plate, 50 μL of the sample was added to 200 μL of solution of DPPH in methanol (100 μ ^m^ ). The mix was incubated in the dark for 30 min. A UV–Vis spectrophotometer (SPECTROstarNano BMG Labtech) was used to measure absorbance at 515 nm on a UV–VIS spectrophotometer (SPECTROstar Nano BMG Labtech). Results are shown as mmol of Trolox Equivalent per kg of extract (mmol TE·kg^−1^ DW). The DPPH assay was performed in triplicate.

### Ferric reducing antioxidant power

The reducing power was determined by ferric reducing antioxidant power (FRAP). The test is based on the ability of antioxidants to reduce ferric ions (Fe^3+^) to ferrous ions (Fe^2+^) through a redox reaction, thus offering a quick and straightforward means of estimating the cumulative reducing power of antioxidants in a sample [20]. The FRAP reagent was prepared by mixing 38 mm of sodium acetate anhydrous buffer in distilled water, pH 3.6, with 20 mm FeCl_3_·6H_2_O in distilled water and 10 mm of 2,4,6‐tripyridyl‐s‐triazine (TPTZ) in 40 mm HCl (10 : 1 : 1). FRAP reagent (180 μL) and 20 μL of sample, or methanol as a blank, were mixed in a 96‐well plate and incubated for 40 min at 37 °C in darkness. Absorbance was measured at 593 nm on a UV–VIS spectrophotometer (SPECTROstar Nano BMG Labtech). Trolox was used as a reference standard antioxidant, and results were expressed as mmol TE·kg^−1^ DW. FRAP assay was performed in triplicate for all samples [18].

### 2,2′‐Azino‐bis (3‐ethylbenzothiazoline‐6‐sulfonic acid)

The ABTS assay is a test based on the ability of antioxidants to scavenge ABTS radicals (ABTS^·+^) generated by the reaction of ABTS with a suitable oxidising agent, such as potassium persulfate [21]. ABTS was dissolved in deionised water to a 7 mm concentration and its radical cation (ABTS^·+^) was produced by reacting ABTS solution with 2.45 mm potassium persulfate and allowing the mixture to stand in the dark at room temperature for 12–16 h before use. Each extract (15 μL) was added to 235 μL of ABTS^·+^ solution and, after 2 h of incubation in the dark, the absorbance was read at 734 nm on a UV–VIS spectrophotometer (SPECTROstar Nano BMG Labtech). All solutions were freshly prepared for the analysis. Results are expressed as mmol TE·kg^−1^ DW [22].

### Cell culture and sample preparation

Human hair follicle dermal papilla cells (HFDPCSs) were purchased from PromoCell (Heidelberg, Germany) and maintained in a follicle dermal papilla cell basal medium supplemented, according to the company, with fetal calf serum (4%), bovine pituitary extract (0.4%), basic fibroblast growth factor (FGF) (1 ng·mL^−1^) and recombinant human insulin (5 μg·mL^−1^) at 37 °C in a humidified atmosphere of 5% CO_2_. For the experiments, the product AT HAIR‐FUL AA^®^ was dissolved in the complete cell culture medium.

### Cell viability assay and protective effect against hydrogen peroxide‐induced oxidative stress

Cell viability was determined using an MTT assay through which it is possible to measure the cells' ability to convert MTT (3‐(4,5‐dimethylthiazol‐2‐yl)‐2,5‐diphenyltetrazolium bromide) into a blue formazan product using their mitochondrial enzymes. The formazan product intensity is directly proportional to the number of viable cells [23]. HFDPCs were seeded into 96‐well plates at a density of 1.5 × 10^4^ cells per well and cultured to subconfluence. The cells were treated with AT HAIR‐FUL AA^®^ extract (0.25–2.00 mg·mL^−1^), PB2 (0.02–0.16 μg·mL^−1^) and chlorogenic acid (0.13–1.05 μg·mL^−1^) for 4, 24, 48, 72 or 96 h. Quantities of PB2 and CGA used were those present in the extract at the respective concentration. The cytoprotective effect exerted by the Annurca extract on HFDPC was evaluated under oxidative stress induced by hydrogen peroxide (H_2_O_2_) (400 μm). After seeding, cells were treated with different concentrations of H_2_O_2_ (100–700 μm) for 2 h. It was found that 400 μm of H_2_O_2_ reduced the cell viability by 50%, which was used for further analysis. Afterwards, HFDPCs were treated with different concentrations of AT HAIR‐FUL AA^®^, either: (a) cells were treated with H_2_O_2_ 400 μm for 2 h and then with the extract for 24 h, or (b) cells were treated for 24 h with extract and then with H_2_O_2_ 400 μm for 2 h [24]. Subsequently, cells were preincubated with PB2 (0.16 μg·mL^−1^) and chlorogenic acid (1.05 μg·mL^−1^) for 24 h and then treated with H_2_O_2_ 400 μm for 2 h. The cells were incubated with MTT (0.75 mg·mL^−1^) for 4 h at 37 °C with 5% CO_2_. MTT is a water‐soluble tetrazolium salt that is metabolised to purple formazan crystals by NAD(P)H‐dependent oxidoreductase enzymes of metabolically active cells. The product was then dissolved in 1 : 1 DMSO : isopropanol solution and spectrophotometrically quantified at 560 nm using a UV–Vis spectrophotometer (SPECTROstarNano BMG Labtech).

### Intracellular reactive oxygen species measurement

The intracellular level of ROS was determined using 2′,7′‐dichlorofluorescein diacetate (DCFH‐DA), a cell‐permeable fluorogenic probe, commonly used to measure intracellular ROS levels in cells. DCFH‐DA is a nonfluorescent compound that can passively diffuse into cells where is cleaved by intracellular esterases to form DCFH (2′,7′‐dichlorofluorescin), which is then oxidised by ROS to produce the fluorescent compound DCF (2′,7′‐dichlorofluorescein).

Briefly, 1 × 10^5^ cells were seeded into 24‐well plates and, after reaching subconfluence, were incubated with AT HAIR‐FUL AA^®^ (2 mg·mL^−1^), PB2 (0.16 μg·mL^−1^) and CGA (1.05 μg·mL^−1^) for 24 h and then stressed with H_2_O_2_ 400 μm for 2 h. The antioxidant molecule NAC 10 mm was used as a positive control. Cells were stained with 10 μm DCFH‐DA for 30 min in the dark at 37 °C in a humidified 5% CO_2_ atmosphere. The cells were then trypsinised, washed with phosphate‐buffered saline (PBS) and analysed by BD FACS Canto II flow cytometer (BD Pharmingen, San Jose, CA, USA) (λex 485 nm and λem 515–540 nm) [23].

### Western blotting

The HFDPCs were seeded in 6‐well plate (3 × 10^5^ cells per well) and treated with AT HAIR‐FUL AA^®^ (2 mg·mL^−1^), PB2 (0.16 μg·mL^−1^) and CGA (1.05 μg·mL^−1^) for 24 h, with or without stress with H_2_O_2_ 400 μm for 2 h. HFDPCs were washed with ice‐cold PBS and lysed in RIPA buffer supplemented with Protease Inhibitor Cocktail. Following centrifugation at 13 000 ***g*** for 10 min, the supernatant was prepared as a protein extract. Protein concentrations were determined using a Bradford reagent. Equal amounts of protein from each sample were mixed with a sample buffer and separated using Bolt™ 4–12% Bis‐Tris Plus gels. The separated proteins were transferred to a nitrocellulose membrane. The membranes were blocked with 5% (weight per volume) nonfat dried milk and 0.025% Tween‐20 in PBS (PBS‐T) and incubated for 1 h at room temperature. Then, the membranes were probed overnight at 4 °C with specific primary antibodies: anti‐SOD2 1 : 1000 (Cat. no.: PA1‐31072; Thermo Fisher Scientific, Waltham, MA, USA); anti‐CAT 1 : 1000 and anti‐β‐actin 1 : 5000 (Cat. no.: 12980S and 4970S; Cell Signaling Technology, Danvers, MA, USA); FGF‐2 1 : 1000 (Cat. no.: A40635; Antibodies.com, Stockholm, Sweden and St Louis, MO, USA); β‐catenin 1 : 1000 (Cat. no.: 06‐734; Sigma). After incubation, the membranes were washed three times with PBS‐T for 10 min and incubated with the appropriate horseradish peroxidase‐conjugated secondary antibody (anti‐rabbit and anti‐mouse 1 : 5000 Cat. no.: A282607 and A17352; Antibodies.com) at room temperature for 1 h. The membranes were washed three times with PBS‐T and the signals visualised by Super Signal West Femto Maximum Sensitivity Substrate (Thermo Fisher Scientific), using the Chemidoc XRS detection system equipped with image lab Software for image acquisition (Bio‐Rad, Hercules, CA, USA) [23]. The quantification of protein bands was carried out by the assessment of the relative optical density using imagej 1.52a software (National Institute of Health) [25].

### Statistical analysis

All experiments were carried out in triplicate and results were expressed as mean ± standard deviation (SD). Statistical analysis was performed using GraphPad Prism 5 Software, Inc. (San Diego, CA, USA). One‐way ANOVA tests were performed with the Tukey–Kramer *post hoc* test, and *P*‐values ≤ 0.05 were considered as statistically significant.

## Results and Discussion

### HPLC‐DAD analysis

**Fig. 1:**
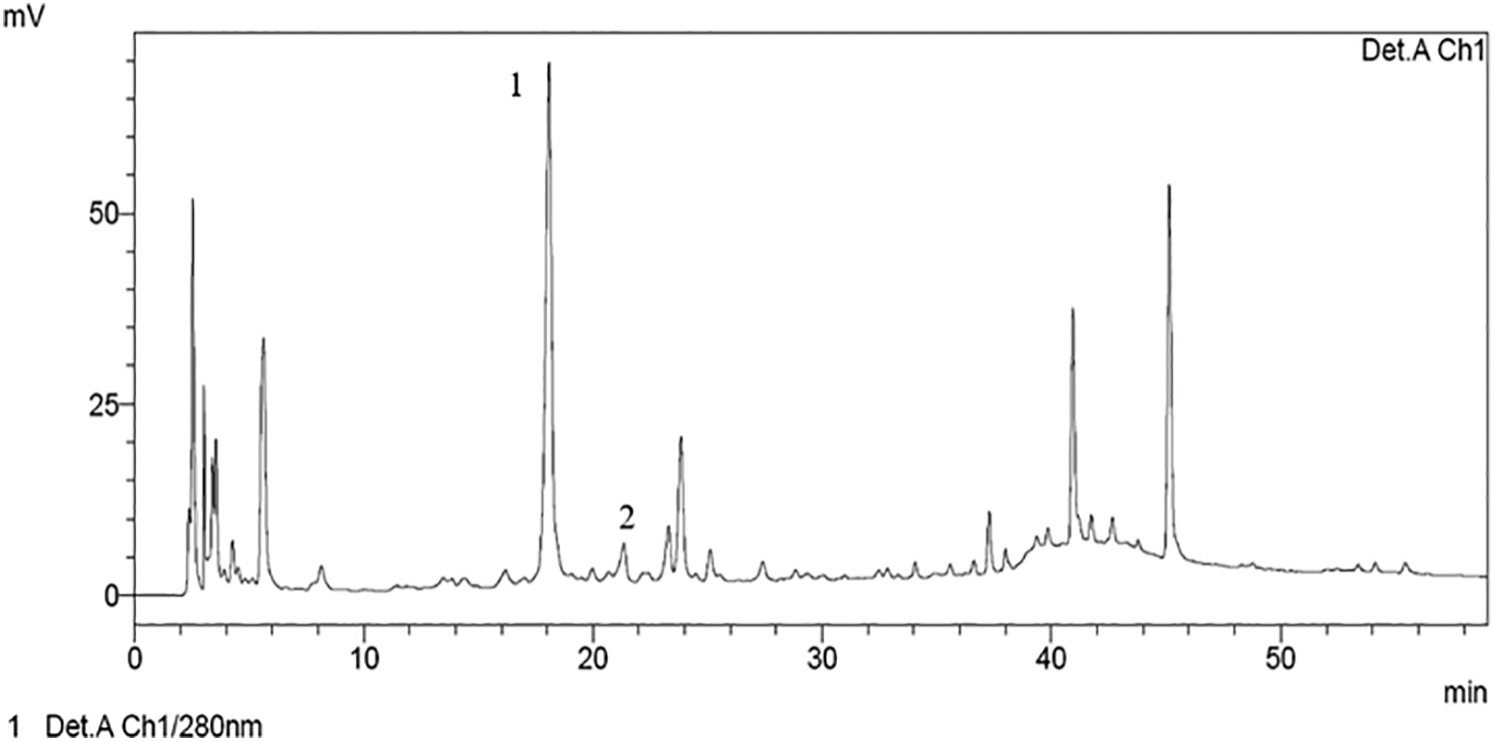
Representative chromatogram of AT HAIR‐FUL AA®. (1) Chlorogenic acid; (2) procyanidin B2. The analysis was performed using a wavelength of 280 nm.

The two main phenolic compounds involved in preventing PHL are procyanidin B2 and chlorogenic acid. They may stimulate hair epithelial cell growth and induce the anagen phase to almost the same extent as MIN [8]. Specifically, *in silico* investigations have demonstrated that chlorogenic acid may bind the catalytic site of the androgen receptor, which is known to play a vital role in controlling hair growth, with a higher affinity than MIN [26]. The amount of procyanidin B2 or chlorogenic acid in AT HAIR‐FUL AA^®^ dry extract was determined using HPLC‐DAD. AT HAIR‐FUL AA^®^ dry extract contained 0.080 ± 0.0086 and 0.523 ± 0.019 mg·g^−1^ of procyanidin B2 and chlorogenic acid, respectively (Fig. 1).

The data obtained are similar to those of a previous study conducted on lyophilised Annurca apples subjected to acidic treatment to enhance phenol extraction (0.088 ± 0.003 and 0.521 ± 0.051 mg·g^−1^ of procyanidin B2 and chlorogenic acid, respectively) [27], indicating that maceration with only water may be a suitable alternative for the extraction of these compounds.

### Total phenolic content and antioxidant activity

The occurrence of various human illnesses, including heart disease, metabolic disorders, cancer and ageing, can be attributed to the involvement of free radicals and oxidative stress, which may act through multiple cellular mechanisms. Recent evidence has highlighted the role of oxidative stress in hair loss processes. Several studies indicated that natural compounds such as phenolic molecules and vitamins play a significant role in reducing oxidative stress, attracting attention due to their safety and potential acceptance by patients as nonmedicinal interventions [28]. Apples are known to contain about 400 mg of total phenols per apple, with an antioxidant activity comparable to 1500 mg of pure Vitamin C. Specifically, it has been demonstrated that Apples are rich in flavonoids, including quercetin, procyanidins, anthocyanidins, (+)‐catechin and (−)‐epicatechin. They also contain dihydrochalcones, such as phlorizin and phloretin, and other polyphenolic compounds, such as chlorogenic acid [13]. In light of this, it was decided to determine the TPC of AT HAIR‐FUL AA^®^ by using the Folin–Ciocâlteu assay. This assay is widely used in nutritional and clinical investigations as it allows the TPC and antioxidant activity in a plant extract and a biological sample to be defined contemporaneously. AT HAIR‐FUL AA^®^ had a TPC of 4.91 ± 0.24 mg GAE·g^−1^, higher than the TPCs of 1.94 ± 0.03 mg GAE·g^−1^ [29] and 2.87 ± 0.12 mg GAE·g^−1^ [27] previously reported in the literature for the whole Annurca fruit. The use of different extractive solvents, methods, stages of apple maturation and treatments may explain variations in free phenolic content among studies. For example, it has been demonstrated that the reddening treatment may be responsible for bioactive compound concentration and, consequently, the different antioxidant activity of Annurca fruit [14]. However, a similar result to that reported here was achieved after using an aqueous‐methanol extraction of Annurca pomace (5.56 mg GAE·g^−1^) [30]. However, it is known that methanol, although it has a high extraction power for bioactive molecules from plant matrices, is not a suitable solvent for obtaining extract destined for nutraceutical production due to its toxicity. In the present investigation, using water, TPC values similar to those from methanol extraction were obtained, indicating that a safe solvent such as water may also be employed for extracting phenolic compounds from Annurca apples.

It is commonly acknowledged that evaluating the antioxidant activity of an extract requires more than a single test. Antioxidants consist of a varied range of substances, and each compound, depending on its chemical structure, may have specific capabilities to counteract certain types of radicals or oxidisers. Therefore, employing multiple techniques is essential to obtaining a comprehensive and precise understanding of the extract's antioxidant characteristics [18]. Table 1 shows the results of the FRAP, DPPH and ABTS assays.

**Table 1:** Antioxidant activity of AT HAIR‐FUL AA®. Experiments were carried out in triplicate, and data were reported as mean ± standard deviation (SD).

	TPC^a^ (mg GAE·g^−1^ DW)	DPPH^b^ (mmol TE·kg^−1^ DW)	ABTS^c^ (mmol TE·kg^−1^ DW)	FRAP^d^ (mmol TE·kg^−1^ DW)
AT HAIR‐FUL AA®	4.91 ± 0.24	19.35 ± 0.48	15.92 ± 0.94	26.60 ± 0.65

^a^ Total phenolic content expressed as milligrams of gallic acid per gram of extract

^b^ 2,2‐Diphenyl‐1‐picrylhydrazyl expressed as milligrams of Trolox equivalents per gram

^c^ 2,2′‐Azino‐bis(3‐ethylbenzothiazoline‐6‐sulfonic acid)

^d^ Ferric reducing antioxidant power expressed as milligrams of Trolox equivalents per grams of extract.

In this investigation, the Annurca apple extract AT HAIR‐FUL AA^®^ showed a reducing ability of 19.35 ± 0.48 mmol TE·kg^−1^ DW, which is higher than that reported in a previous study (15.59 ± 0.11 mmol TE·kg^−1^ DW) [27]. The ability of the extract to scavenge the ABTS radicals was tested, obtaining an ABTS value of 15.92 ± 0.94 mmol TE·kg^−1^ DW was obtained. Finally, the FRAP assay is commonly employed to assess the overall antioxidant capacity of biological samples, food products and natural extracts [30]. AT HAIR‐FUL AA^®^ dry extract had an antioxidant activity evaluated with FRAP of 26.60 ± 0.65 mmol TE·kg^−1^ DW. This is similar to previously reported data (FRAP value of 26.63 ± 0.70 mmol TE·kg^−1^ DW) [27]. It is of note that, despite the different extractive methods used in the current study and by Maisto *et al*. [27], the same FRAP‐reducing power result was obtained. Maisto *et al*. [27] treated the Annurca whole fruit with methanol/water (90 : 10, v/v) as a solvent and used ultrasound‐assisted extraction (UAE). Ultrasound‐assisted extraction is a commonly employed method for extracting secondary metabolites from plants. It utilises high‐frequency ultrasound waves to enhance the extraction process by facilitating mass transfer and cell disruption. By applying ultrasound waves, this method promotes the release of target compounds from the plant material, leading to increased extraction efficiency. Several investigations have used UAE for extracting Annurca apple specialised metabolites [27, 29, 31]; however, this method is not relevant for extract production on an industrial scale [32], as the equipment to be used would be too expensive. Hence, in this study, for the preparation of AT HAIR‐FUL AA^®^, we used maceration as the process is industrially scalable and does not require excessive energy expenditure, as in the case of the UAE. As previously stated, the most commonly used solvent for phenols extraction is methanol; however, it is toxic and so not suitable for the preparation of nutraceutical formulations for human use. For this reason, in the present investigation, water was used as the extractive solvent for preparing AT HAIR‐FUL AA^®^, still obtained antioxidant activity and TPC results similar to or higher than those previously reported in the literature.

### Effect of AT HAIR‐FUL AA^®^ extract on cell viability and oxidative stress‐induced cell death assay

HFDPCs were used as cell models to evaluate the cytotoxic effect of AT HAIR‐FUL AA^®^. They are a specialised cell type located in the dermal papilla of hair follicles: A unique structure situated at the base of the hair follicle, and they play a crucial role in hair growth and hair cycle regulation. Specifically, HFDPCs provide signals and support to the surrounding cells within the hair follicle, including the hair matrix cells responsible for producing hair fibres. HFDPCs are, therefore, extensively used in the study of hair biology and generation [33]. The viability of HFDPCs was evaluated by MTT assay after treating cells with different concentrations of AT HAIR‐FUL AA^®^ (0.25–2.00 mg·mL^−1^) from 4 to 96 h. Procyanidin B2 (0.16–0.02 μg·mL^−1^) and chlorogenic acid (0.13–1.05 μg·mL^−1^) were used as reference compounds according to the quantification carried out by HPLC‐DAD analysis. The extract and the pure molecules showed no cytotoxic effect on HFDPCs at tested concentrations, and the study moved on.

**Fig. 2:**
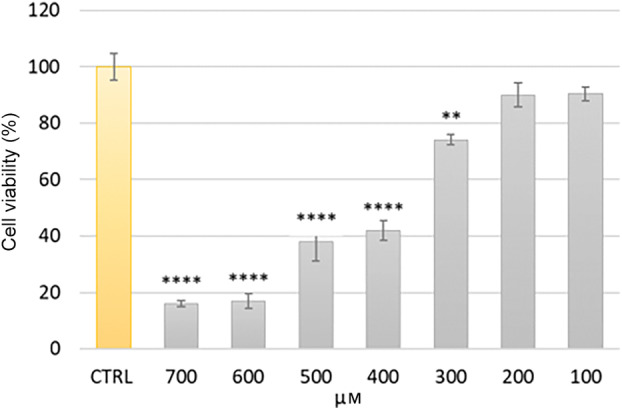
Cell viability of human hair follicle dermal papilla cells (HFDPCs) treated with H_2_O_2_. Cells were treated with different concentrations (100–700 μm) of H_2_O_2_ for 2 h. Cell viability was evaluated by MTT assay. Data are expressed as the mean ± SD of three independent experiments (*n* = 3). **P < 0.01, ****P < 0.0001.

Before determining the extract's ability to prevent or avoid cell death by oxidative stress, the hydrogen peroxide (H_2_O_2_) concentration range that induced cell death due to oxidative stress was identified as a preliminary test. HFDPCs were treated with different concentrations of H_2_O_2_ (100–700 μm) for 2 h and cell viability was evaluated by MTT assay. Compared with the control group, H_2_O_2_ caused significant cytotoxicity at 400 μm (*P* < 0.0001) (Fig. 2), which is similar to findings in another study [34]. Subsequent tests therefore used a concentration of 400 μm H_2_O_2_ to assess the ability of AT HAIR‐FUL AA^®^ to suppress HFDPCs death induced by oxidative stress.

**Fig. 3:**
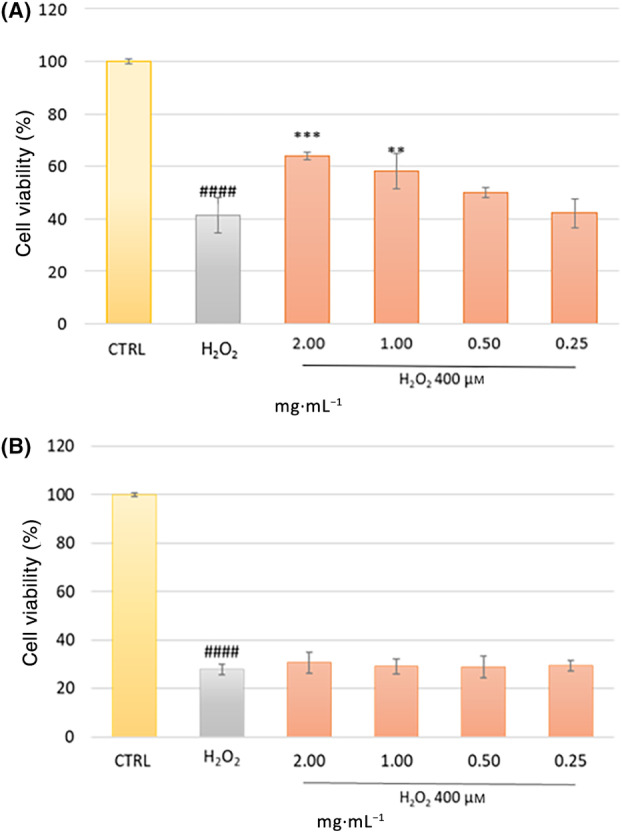
Evaluation of the protective effect of AT HAIR‐FUL AA® against H_2_O_2_‐induced oxidative stress. Human hair follicle dermal papilla cells (HFDPC) were (A) preincubated with different concentrations of AT HAIR‐FUL AA® (0.25–2 mg·mL^−1^) for 24 h and then treated with H_2_O_2_ 400 μm for 2 h. (B) Cells were preincubated with H_2_O_2_ 400 μm for 2 h and then treated with different concentrations of AT HAIR‐FUL AA® (0.25–2 mg·mL^−1^). Cell viability was evaluated by MTT assay. Data are expressed as mean ± SD of three independent experiments (*n* = 3). ^####^ *P* < 0.0001 *vs* CTRL. **P < 0.01, ***P < 0.001, *vs* H_2_O_2_‐treated group.

**Fig. 4:**
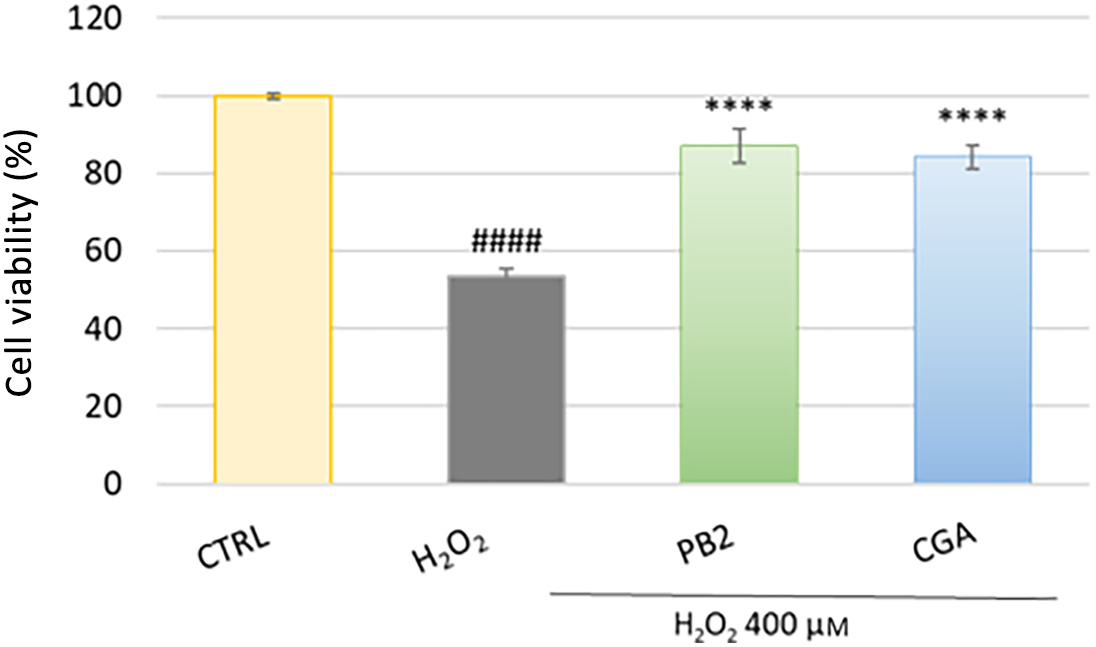
Evaluation of the protective effect of procyanidin B2 and chlorogenic acid against H_2_O_2_‐induced oxidative stress. Human hair follicle dermal papilla cells (HFDPC) were preincubated with procyanidin B2 (PB2, 0.16 μg·mL^−1^) and chlorogenic acid (CGA, 1.05 μg·mL^−1^) for 24 h and then treated with H_2_O_2_ 400 μm for 2 h. Cell viability was evaluated by 3‐(4,5‐dimethyl‐2‐thiazolyl)‐2,5‐diphenyl‐2H‐tetrazolium bromide dye (MTT) assay. Data are expressed as mean ± SD of three independent experiments (*n* = 3). ^####^ *P* < 0.001 *vs* CTRL group and ****P < 0.0001 *vs* H_2_O_2_‐treated group.

To evaluate this preventive effect, HFDPCs were pretreated with the extract for 24 h before stress exposure for 2 h. AT HAIR‐FUL AA^®^ demonstrated the ability to prevent the loss of viability caused by H_2_O_2_ in a dose‐dependent manner (Fig. 3A), showing the highest activity at 2 mg·mL^−1^. In light of this result, the concentration of 2 mg·mL^−1^ was chosen for further experiments. However, the extract could not protect the cells when used with a curative intention following the oxidative stress induced by H_2_O_2_ (Fig. 3B). To date, this is the first study evaluating the cytoprotective effect of Annurca apple extract on oxidative stress‐induced‐HFDP cell death. This protective effect may be due to the presence of phenolic compounds, as demonstrated by the ability of PB2 and CGA to prevent H_2_O_2_‐induced cell death (Fig. 4).

### AT HAIR‐FUL AA® attenuated the H_2_O_2_‐induced increase in ROS level in HFDPCs

**Fig. 5:**
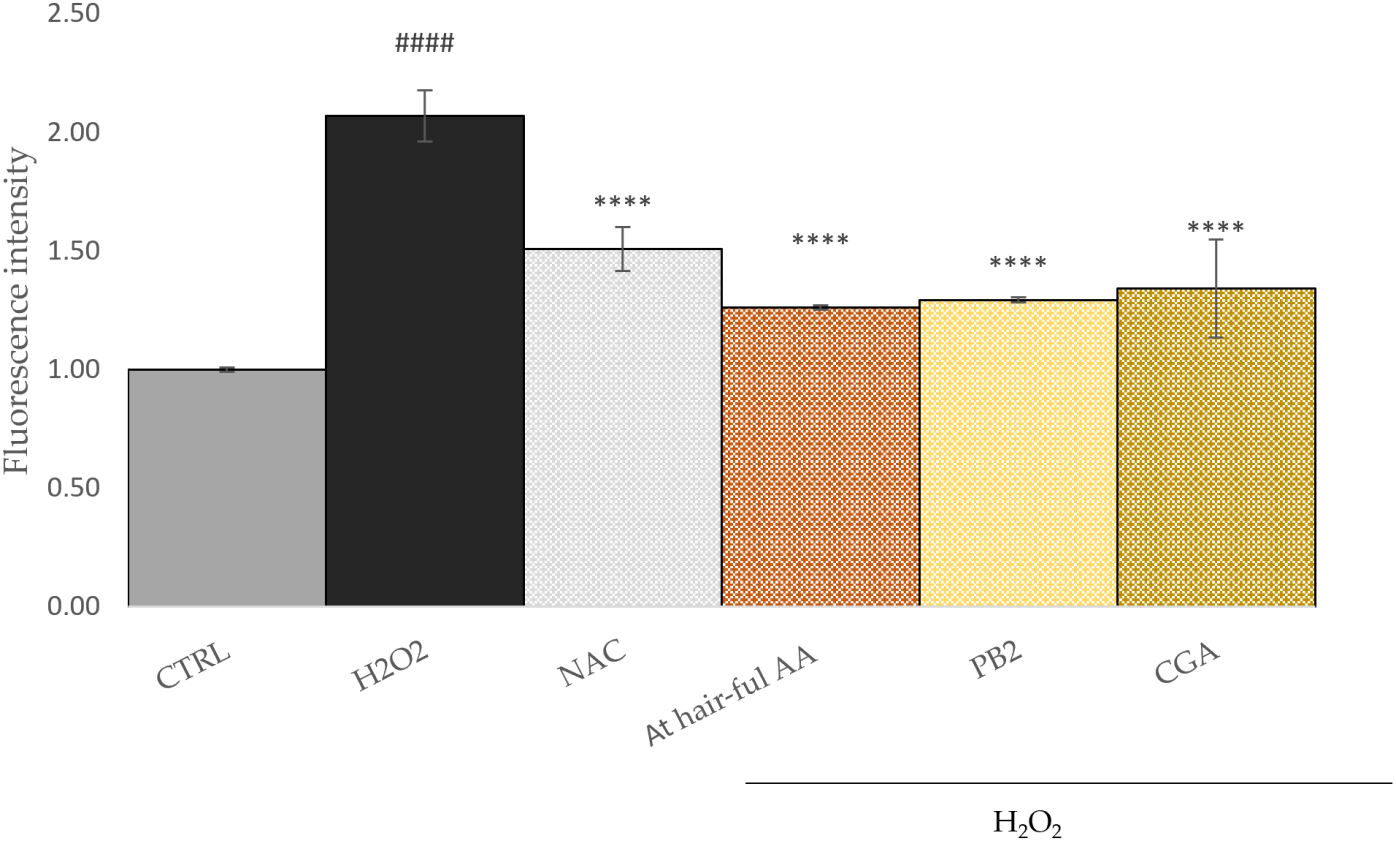
Human hair follicle dermal papilla cells (HFDPC) were pretreated with AT HAIR‐FUL AA® (2 mg·mL^−1^), procyanidin B2 (PB2, 0.16 μg·mL^−1^) and chlorogenic acid (CGA, 1.05 μg·mL^−1^), and *N*‐acetyl cysteine (NAC) for 24 h, and then treated with or without H_2_O_2_ 400 μm for 2 h. ROS generation was measured by flow cytometry using 2′,7′‐dichlorodihydrofluorescein diacetate (DCFH‐DA) staining. Data are expressed as the mean ± SD of three independent experiments (*n* = 3). ^#^^###^ *P* < 0.0001 *vs* CTRL and ****P < 0.0001 *vs* H_2_O_2_‐treated cells.

After demonstrating the ability of AT HAIR‐FUL AA^®^ to prevent oxidative stress‐induced cell death, the H_2_O_2_‐mediated accumulation of intracellular ROS was determined. HFDPCs were pretreated with AT HAIR‐FUL AA^®^ (2 mg·mL^−1^) for 24 h, and then, oxidative stress was induced with H_2_O_2_. ROS generation was quantified using the fluorogenic probe DCFH‐DA. AT HAIR‐FUL AA^®^ did not alter intracellular ROS levels in basal conditions (data not shown), but it significantly reduced H_2_O_2_‐induced intracellular ROS levels in the same manner as the *N*‐acetyl cysteine (NAC) used as a positive control (Fig. 5).

AT HAIR‐FUL AA^®^ did not appear to alter the basal levels of intracellular ROS. This is a positive result, as it is known that they play a crucial role in physiological conditions. ROS are key signalling molecules involved in various cellular processes such as cellular signalling, immune response, cellular metabolism, hypoxia signalling and cellular homeostasis [35]. However, it is worth noting that the balance between ROS generation and antioxidant defence mechanisms is crucial for maintaining the physiological role of ROS. Excessive ROS production or insufficient antioxidant capacity can lead to oxidative stress, which is associated with various pathological conditions, including cardiovascular disorders, neurodegenerative diseases, ageing and hair loss. Therefore, regulating ROS levels is essential for preventing cellular damage and maintaining physiological processes. AT HAIR‐FUL AA^®^ may have a role in ensuring the redox state balance, as a reduction in intracellular ROS in stress conditions was observed. To date, this is the first study evaluating the potential ability of Annurca apple extract to counteract oxidative stress in HFDPCs. However, previous investigations have demonstrated the protective effect of Annurca against ROS on other cell lines, such as anaplastic thyroid cancer cells (CAL62) [36] and mammary epithelial cells (MCF10A) cells [37]. These results indicate that AT HAIR‐FUL AA^®^ can impede H_2_O_2_‐treated HFDPC ROS accumulation and apoptosis and, therefore, may be used as a potential supplement for preventing hair loss induced by oxidative stress.

### AT HAIR‐FUL AA^®^ modulates the protein expression of antioxidant enzymes

**Fig. 6:**
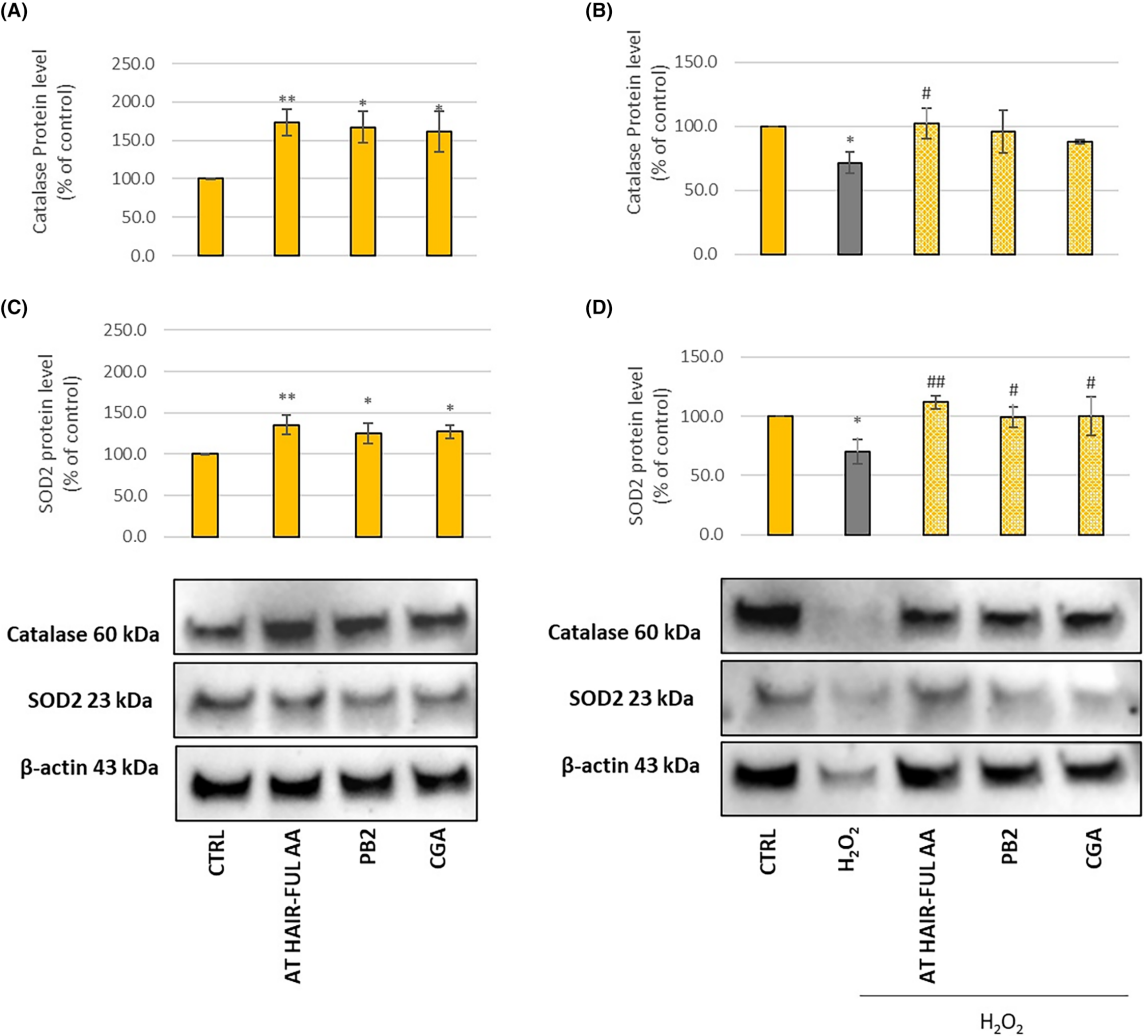
Effect of AT HAIR‐FUL AA® on antioxidant enzymes. Human hair follicle dermal papilla cells (HFDPCs) treated with AT HAIR‐FUL AA® (2 mg·mL^−1^), procyanidin B2 (PB2 0.16 μg·mL^−1^) and chlorogenic acid (CGA 1.05 μg·mL^−1^) (A–C); HFDPCs were stressed with H_2_O_2_ 400 μm for 2 h and then treated with AT HAIR‐FUL AA® (2 mg·mL^−1^), procyanidin B2 (PB2, 0.16 μg·mL^−1^) and CGA (1.05 μg·mL^−1^) (B–D) The protein levels were normalised with β‐actin content. Data were normalised to control cells set to 100% and expressed as the mean ± SD of three independent experiments (*n* = 3). *P < 0.05, **P < 0.01 *vs* CTRL group and ^#^ *P* < 0.05, ^##^ *P* < 0.01 *vs* H_2_O_2_‐treated group.

To assess whether AT HAIR‐FUL AA^®^ can maintain the redox status in HFDPCs, its effect on antioxidant enzyme expression, including SOD2 and CAT, in basal conditions or H_2_O_2_‐induced oxidative stress was investigated. SOD2, also known as manganese superoxide dismutase (MnSOD), is a superoxide dismutase (SOD) isoform that specifically exists within the cells' mitochondria. This enzyme is implicated in neutralising superoxide radicals and protecting cells from oxidative damage. It has been demonstrated that SOD2 mutations are associated with several age‐related disorders; hence, its role in maintaining mitochondrial health and cellular redox balance is crucial for overall cellular function and well‐being [38, 39]. Catalase (CAT) is another crucial antioxidant enzyme involved in protecting cells from oxidative stress. CAT activity is crucial for maintaining cellular redox balance by working in coordination with other antioxidant enzymes, such as SOD, to neutralise ROS and maintain cellular homeostasis [40]. Free radical production increases with age and there is a concomitant reduction in the function of endogenous defence mechanisms. This leads to the progressive damage of cellular structures. Specifically, in the context of hair, the increase in oxidative stress results in greying due to impaired melanocyte function, reduced hair production and alopecia [41]. A clinical trial conducted on 15 elderly participants in Turkey demonstrated that fresh apple consumption (2 g·kg^−1^ for 1 month) increased subjects' plasma and erythrocyte antioxidant activity. This increase was attributed to enhanced antioxidant enzyme activity, such as SOD, in erythrocytes [42]. We therefore decided to investigate the ability of AT HAIR‐FUL AA^®^ to increase the expression of SOD2 and CAT in HFDPCs in basal conditions or H_2_O_2_‐induced oxidative stress. Protein expression was measured by western blot upon treatment with AT HAIR‐FUL AA^®^ (2 mg·mL^−1^) or procyanidin B2 (0.16 μg·mL^−1^) or chlorogenic acid (1.05 μg·mL^−1^) for 24 h, demonstrating that the expression of both enzymes was increased by treatment with either AT HAIR‐FUL AA^®^, procyanidin B2 and chlorogenic acid (Fig. 6A,C). When stressed cells were treated with AT HAIR‐FUL AA^®^, an increase in SOD2 and CAT expression (Fig. 6B,D) was observed compared with the control (H_2_O_2_, 400 μm) (*P* < 0.05 and *P* < 0.01 respectively). The active metabolites PB2 and CGA, upregulated the SOD2 enzyme (Fig. 6D) and restored CAT expression to basal levels (Fig. 6C), as did the Annurca extract. Interestingly, in stressed conditions, AT HAIR‐FUL AA^®^ increased CAT expression more than the single compounds, indicating a possible synergy of Annurca specialised metabolites (Fig. 6C). This observation accords with the knowledge that an increase in dietary fresh fruit intake brings more health effects than a single dietary supplement due to the possible synergic effect of fruit antioxidant micronutrients and macronutrients.

These data corroborated the results of an *in vivo* investigation showing that a diet rich in Annurca apple given to aged rats restored hippocampus SOD activity to the levels of young animals [15].

### Growth factor expression in AT HAIR‐FUL AA^®^‐treated HFDPC

**Fig. 7:**
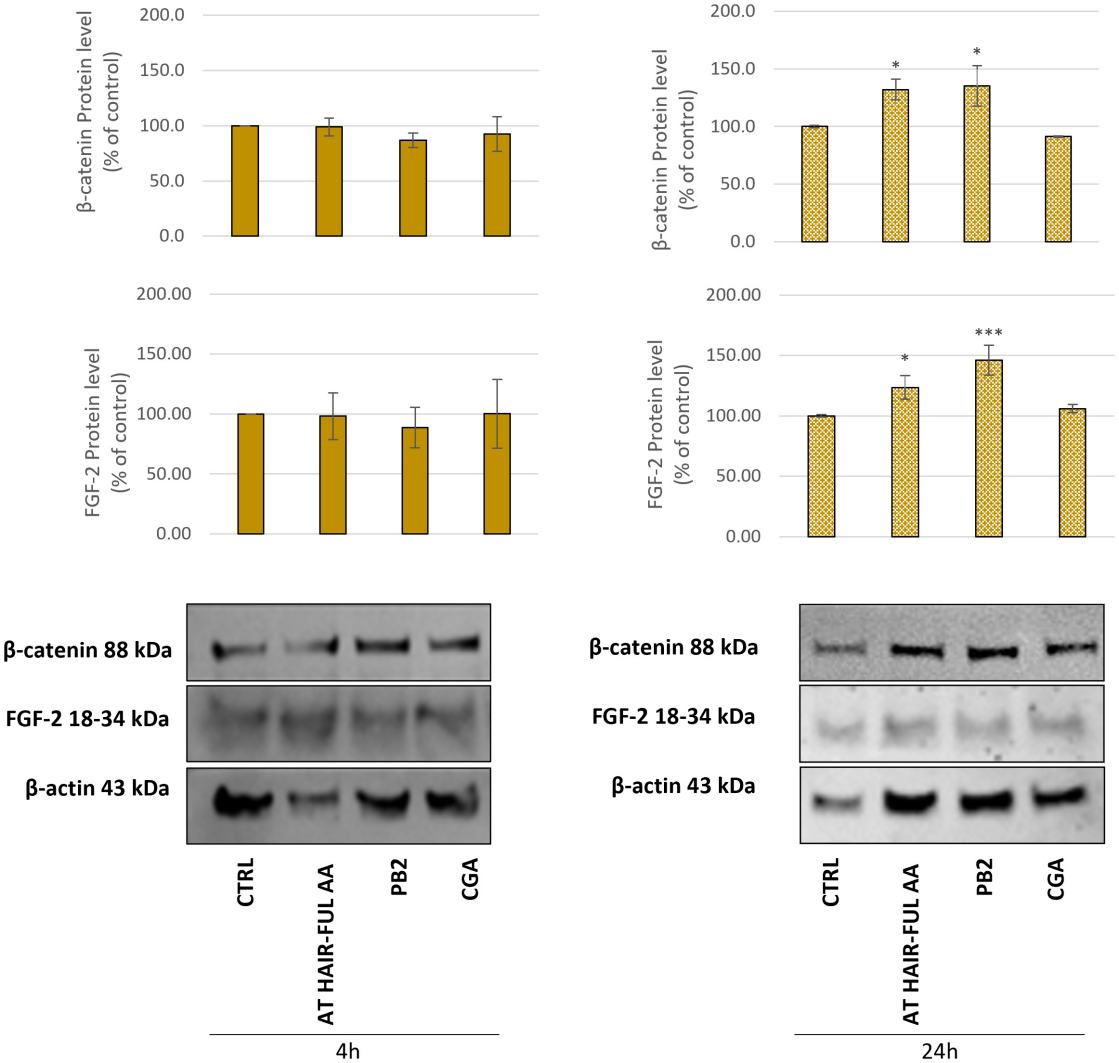
Effect of AT HAIR‐FUL AA® on hair growth factors. Human hair follicle dermal papilla cells (HFDPCs) were treated with AT HAIR‐FUL AA® (2 mg·mL^−1^) or procyanidin B2 (PB2, 0.16 μg·mL^−1^) or chlorogenic acid (CGA, 1.05 μg·mL^−1^) for 4 h and 24 h. The protein levels were normalised with β‐actin content. Data were normalised to CTRL group, which was set to 100% and expressed as the mean ± SD of three independent experiments (*n* = 3). *P < 0.05, ***P < 0.001.

β‐Catenin is the main factor of the Wingless/Integrated (Wnt) signalling pathway, which is involved in the regulation of epithelial cell differentiation, leading to the formation of hair follicles and the generation of the hair shaft and root inner sheath [43]. It has been shown that the knockdown of β‐catenin prevents stem cells from differentiating into follicular keratinocytes, thereby impairing the morphogenesis and regeneration of hair follicles [44]. β‐Catenin is, therefore, the responsible factor for promoting the hair cycle transition from the telogen to the anagen phase [43]. Considering the importance of this factor in hair growth, we investigated the role of AT HAIR‐FUL AA^®^ and its main metabolites in enhancing β‐catenin expression in HFDPCs after 4 and 24 h by using western blot. Compared with the control group, β‐catenin was significantly increased after 24 h of treatment with the extract and PB2 (*P* < 0.05) (Fig. 7).

Previous *in vivo* administration of Annurca apple extract to mice resulted in an improvement of hair length, weight, thickness and density by increasing the expression of the FGF and vascular endothelial growth factor A (VEGFA) [45]. These two factors are known to be induced by the Wnt/β‐catenin pathway, indicating a possible involvement of Annurca extract in enhancing canonical Wnt signalling activation via increased β‐catenin expression [45]. This assumption was corroborated by an observed increase in FGF‐2 expression in HFDPCs following treatment with AT HAIR‐FUL AA^®^. Similarly, PB2 also induced an increase in FGF‐2 expression compared with nontreated cells (***P < 0.001) (Fig. 7). This is an interesting result, as FGF plays a key role in the development of hair follicles and the differentiation and proliferation of epidermal cells [46]. It has been demonstrated that FGF‐2 has a positive influence on mice's hair growth cycle [47].

The ability of AT HAIR‐FUL AA^®^ to induce the expression of β‐catenin and follicular growth factors, in addition to its ability to reduce oxidative stress by increasing the expression of antioxidant enzymes, may explain the results obtained in a recent clinical trial involving AT HAIR‐FUL AA^®^. This randomised, double‐blind controlled parallel group trial compared the effect of Annurca apple extract, AT HAIR‐FUL AA^®^ procyanidin B2 whole fruit (peel and pulp) and placebo in patients with androgenic alopecia. It was reported that the administration of two capsules per day of AT HAIR‐FUL AA^®^ decreased hair loss and increased hair weight and density [17]. Hence, considering the data obtained in the present investigation and from the literature, it is possible to state that Annurca apple fruit extract, AT HAIR‐FUL AA^®^, may represent a promising agent usable for inducing hair growth by counteracting oxidative stress and inducing hair growth factors.

## Conclusions

For the treatment of PHL, several pharmacological medications have been developed over the years, but only a small volume of information on their effectiveness has been published. These factors have resulted in the pharmaceutical and medical industries concentrating their efforts on the development of novel and safer treatments. Among these treatments, Annurca extract was found to be able to induce hair growth *in vivo* and in healthy human subjects. In this work, a new Annurca extract, AT HAIR‐FUL AA^®^, was investigated for the first time on HFDPCs, demonstrating its ability to counteract intracellular ROS accumulation by increasing the activity of the antioxidant enzymes SOD and CAT. Furthermore, it demonstrated the implication of AT HAIR‐FUL AA^®^ in increasing β‐catenin, a factor known to be involved in hair follicle morphogenesis and regeneration, promoting the transition from telogen to anagen phase. The production was also associated with increased FGF‐2, which plays a key role in developing hair follicles. Overall, these findings suggest that AT HAIR‐FUL AA^®^ may be a potential therapeutically useful natural substance for preventing or treating hair loss by reducing oxidative stress and inducing the expression of hair growth‐related factors.

## Conflict of interest

The authors declare that this study received funding from EVRA S.r.l. Società Benefit. The company had no role in the design of the study; in the collection, analyses or interpretation of data; in the writing of the manuscript; or in the decision to publish the results.

### Peer review

The peer review history for this article is available at https://www.webofscience.com/api/gateway/wos/peer‐review/10.1002/2211‐5463.13805.

## Author contributions

DR and LM contributed to the conceptualisation and supervision. NB, CM and DR contributed to the methodology, software, formal analysis, investigation and data curation. NB, CM, DR and LM contributed to the validation, writing—original draft preparation, and writing—review and editing. FDB, RFC and LM contributed to the resources and project administration. LM contributed to the funding acquisition. All authors have read and agreed to the published version of the manuscript.

## Data Availability

The data that support the findings of this study are available from the corresponding author (luigi.milella@unibas.it) upon reasonable request.
